# Disparities in allele frequencies and population differentiation for 101 disease-associated single nucleotide polymorphisms between Puerto Ricans and non-Hispanic whites

**DOI:** 10.1186/1471-2156-10-45

**Published:** 2009-08-14

**Authors:** Josiemer Mattei, Laurence D Parnell, Chao-Qiang Lai, Bibiana Garcia-Bailo, Xian Adiconis, Jian Shen, Donna Arnett, Serkalem Demissie, Katherine L Tucker, Jose M Ordovas

**Affiliations:** 1 Jean Mayer US Department of Agriculture Human Nutrition Research Center on Aging, Tufts University, Boston, MA, USA; 2 Friedman School of Nutrition Science and Policy, Tufts University, Boston, MA, USA; 3 Department of Nutritional Sciences, Faculty of Medicine, University of Toronto, Toronto, Ontario, Canada; 4 Genome Biology and Cell Circuits Program, Broad Institute, Cambridge, MA, USA; 5 Department of Epidemiology, University of Alabama, Birmingham, Birmingham, AL, USA; 6 Department of Biostatistics, School of Public Health, Boston University, Boston, MA, USA

## Abstract

**Background:**

Variations in gene allele frequencies can contribute to differences in the prevalence of some common complex diseases among populations. Natural selection modulates the balance in allele frequencies across populations. Population differentiation (F_ST_) can evidence environmental selection pressures. Such genetic information is limited in Puerto Ricans, the second largest Hispanic ethnic group in the US, and a group with high prevalence of chronic disease. We determined allele frequencies and population differentiation for 101 single nucleotide polymorphisms (SNPs) in 30 genes involved in major metabolic and disease-relevant pathways in Puerto Ricans (n = 969, ages 45–75 years) and compared them to similarly aged non-Hispanic whites (NHW) (n = 597).

**Results:**

Minor allele frequency (MAF) distributions for 45.5% of the SNPs assessed in Puerto Ricans were significantly different from those of NHW. Puerto Ricans carried risk alleles in higher frequency and protective alleles in lower frequency than NHW. Patterns of population differentiation showed that Puerto Ricans had SNPs with exceptional F_ST _values in intronic, non-synonymous and promoter regions. NHW had exceptional F_ST _values in intronic and promoter region SNPs only.

**Conclusion:**

These observations may serve to explain and broaden studies on the impact of gene polymorphisms on chronic diseases affecting Puerto Ricans.

## Background

Close to four million Puerto Ricans live in the United States mainland, representing the second largest Hispanic ethnic group after Mexican-Americans [1]. The few studies on health status or disparities that have focused on Puerto Ricans living on the US mainland consistently show excess chronic health conditions for this population when compared with non-Hispanic whites (NHW) and other Hispanic subgroups [2,3]. In particular, Puerto Ricans exhibit high prevalence of diabetes, heart disease, obesity, depression, and cognitive and functional decline [4-9]; national data points to a 12.6% prevalence of diabetes in Puerto Ricans aged 20 years or older [10], while close to 61% of Puerto Rican adults are overweight or obesity [7], and an estimated 9.3% have depression [11].

The etiology of such conditions is multi-factorial, and most likely, a combination of genetic, social and environmental factors define the health disparities observed in Puerto Ricans. Still, studies on the genetic contribution to such conditions in this population are limited. Generalizing genetic risk to Puerto Ricans based on results obtained from other Hispanic groups is inappropriate due to the unique genetic background of this community, which is quite different from other Hispanic populations studied more often [12-14]. Furthermore, extrapolating results from other broadly researched non-Hispanic populations onto Puerto Ricans could lead to inaccurate conclusions [14].

Several reports have discussed the importance of estimating gene allele frequency in epidemiological and genetic association studies [15-17]. Notably, allele frequencies for single nucleotide polymorphisms (SNPs) can vary by ethnic group, sometimes drastically [18]. Spielman *et al*. recently showed that expression of individual genes differed significantly between ethnic populations, and that variation in frequency of specific alleles accounted for some of these differences in gene expression [19]. Such variations in minor allele frequency (MAF), when mapping to or affecting genes pertinent to a given disease, could translate into differences in the prevalence and incidence of disease across ethnic groups [20]. Indeed, several studies have shown differences in allele distribution of SNPs in candidate genes for immune response and lipid metabolism as well as cardiovascular disease and type 2 diabetes, [17,18,20-23]. Myles *et al*. suggest that SNPs with a large difference in risk allele frequency between populations are strong candidates to explain the variations in disease prevalence between those populations [24]. These observations strongly suggest that Puerto Ricans may also carry disease-associated alleles at frequencies different from other ethnic groups, and that such differences could play a role in the health disparities documented in this population.

The level of population differentiation shown by diverse ethnic groups is related to the variations in allele frequency for genetic polymorphisms [20]. Natural selection modulates the balance in allele frequencies across populations. Certain environmental conditions can act through selective pressure to alter the frequency of a genetic variant resulting in population-specific allele frequencies. Genomic regions of an excess of ancestral divergence between disease and non-disease groups indicates selection signals [25], which allows identification of genomic regions that contribute the risk of disease. Alternatively, the fixation index (F_ST_) measure quantifies population differentiation and tests for evidence of selection pressures, with higher values of F_ST _indicating local positive adaptation and lower values suggesting negative or neutral selection [26]. Several studies have shown that differentiation can vary by chromosomal region and by function of the SNP. Olshen *et al*. reported regions in chromosomes 2 and 6 with high local F_ST _values that also differed between ethnic groups [27]. In addition, higher F_ST _estimates have been reported for SNPs in functional or coding regions than in non-functional or non-coding SNPs [26,28]. Barreiro *et al*. have shown that non-synonymous and 5'-UTR SNPs present extreme degrees of differentiation and positive selection [29]. It has been proposed that loci showing high F_ST _values, or exceptional variance, should be given high priority for association studies of complex diseases and studies on local adaptation to environmental effects [26,30]. Positively selected alleles (those with high F_ST _values) in disease-associated SNPs could be responsible for differences between populations in prevalence or progression of disease [24]. Although F_ST _values could denote other forces such as genetic drift, bottleneck or founder effect in the population, it is still a useful measure to identify genomic regions of potential environmental interactions and association with disease. Such measures of genetic diversity within a population of Puerto Ricans have not been reported.

We have estimated the ancestry of the Puerto Rican population as 57.2% European, 27.4% African, and 15.4% Native American [31]. Population admixture could influence allele frequencies and thus disease prevalence. Given the high prevalence of diabetes, hypertension, and obesity in this population, the aim of this study was to examine the differences in allele frequencies of 101 disease-associated genetic variants in genes of pathways involving lipid and glucose metabolism and inflammation response, between Puerto Ricans and a non-Hispanic white population, which has a much lower prevalence of such conditions and comprise the major ancestry population for Puerto Ricans. In addition, we aimed to identify patterns of population differentiation for Puerto Ricans in comparison to NHW.

## Methods

### Study populations

The Puerto Rican sample consisted of unrelated participants of the Boston Puerto Rican Health Study, a cohort of 1020 Puerto Rican subjects at the time of this analysis, designed to study stress, nutrition, health and aging [32]. Eligible subjects were of Puerto Rican descent, between the ages of 45–75, living in the Boston, MA metropolitan area and able to answer interview questions in either English or Spanish. Subjects self-reported their ethnicity. This study obtained Human Investigation Review Board approval from Tufts Medical Center. Participants provided written informed consent.

The NHW sample was comprised of 1506 participants over 18 years of age participating in the Genetics of Lipid Lowering Drugs and Diet Network (GOLDN) Study. Participants were recruited from the ongoing NHLBI Family Heart Study in two genetically homogeneous centers (Minneapolis, MN and Salt Lake City, UT) with predominantly NHW populations. Race was self-reported. The primary aim of the GOLDN study was to characterize the role of genetic and dietary factors on an individual's response to fenofibrate. The detailed design and methodology of the study have been described previously [33]. The study was approved by the Human Studies Committee of Institutional Review Board at the University of Alabama at Birmingham, the University of Minnesota, University of Utah, and Tufts Medical Center. Written informed consent was obtained from all participants. To compare with the Puerto Rican population, only data from 597 subjects ages 45–75 years were selected.

### Selection of genes and SNPs

Genes and SNPs were selected by bioinformatics analysis, based on previously reported associations or knowledge of their role in known biological pathways such as lipid and glucose metabolism and inflammation response. Briefly, SNP selection took a balanced approach of incorporating results of published studies, of bioinformatics-based predictions of putative functional consequence of the two alleles, and of linkage disequilibrium (LD) analysis to diversely and economically sample different genetic blocks. Putative allele-specific function was assessed according to map position within the gene. SNPs in upstream regions and introns were analyzed for altering transcription factor binding sites. Intronic SNPs could also affect mRNA splicing. Non-synonymous SNPs within coding sequences of exons could alter protein sequence, structure and function while synonymous SNPs could call for codons of a different frequency whose effects can be similar to non-synonymous SNPs. Lastly, 3'-UTR SNPs can alter secondary structure which can affect mRNA stability or interactions with small RNAs (e.g., miRNAs). These approaches are detailed elsewhere [34]. A total of 101 autosomal diallelic SNPs from 30 different genes were genotyped. For each SNP, data for the SNP location on the gene and chromosome, function and accession number were obtained from databases published by the National Center for Biotechnology Information [35]. Available information on reported associations with chronic and metabolic conditions was collected by searching the biomedical literature [35]. Only associations for the individual allele or genotype and for healthy adults in the general population were collected, unless otherwise noted. Associations deemed conflicting were not included unless a meta-analysis was available. A list of all genotyped SNPs, with information on chromosome location, variant name, function, and accession number, is included in Additional file 1.

### Genotyping

Buffy coats of nucleated cells were obtained from blood samples and genomic DNA was purified using the QIAamp^® ^DNA Blood Mini kits (*Qiagen, Hilden, Germany*) according to the vendor's recommended protocol. Genotyping was carried out by the 5'-nuclease assay with allele specific TaqMan probes [36]. TaqMan^® ^SNP genotyping assays, either custom (assays-by-design) or pre-designed (assays-on-demand), were purchased from Applied Biosystems (*Applied Biosystems, Foster City, CA*). For each reaction, 10 ng of DNA were diluted and aliquoted into 96-well plates. Rapidplate robotic system was used in adding DNA samples from 96-well plates into 384-well plates. For PCR, the DNA was mixed with Taqman Universal PCR MasterMix, Assay and water using a TECAN robotic system. PCR cycles were (1) 10 min at 95°, (2) 15 seconds at 92° and (3) one minute at 60°; repeating steps 2 and 3 for 50 cycles. Blinded no-template controls and replicates of DNA samples were included in the DNA sample plates and routinely checked by the laboratory technicians. Applied Biosystems 7900 was used for allelic discrimination, and Microsoft^® ^Excel macros were used to create the plate records with allele base codes and for cleaning data including: checking on negative controls to ensure the match of plate record and plate, allele frequency calculation to ensure the match of marker name and assay used, and preparation of data ready for export into database. Microsoft^® ^Access was used for data consolidation to ensure accuracy. Quality control estimated the genotyping error rate as less than 1%. The descriptions for primers, probes, and sequences, as well as ABI assay-on-demand ID, are presented in Additional file 1.

### Statistical analysis

Statistical analyses were performed in SPSS version 15.0 or Microsoft^® ^Excel. Allele frequencies were estimated based on genotype frequencies. The Puerto Rican sample was analyzed first and minor alleles were assigned based on this population; thus Puerto Ricans have no minor allele frequency greater than 0.50. To calculate the expected MAF of each SNP under an admixture model in Puerto Ricans, we first estimated allele frequency contribution from the European ancestry (57%) and African ancestry (27%) [31], based on the allele frequencies of 73 SNP available in HapMap for the CEU and YRI population reported. As allele frequencies of Native American are not available in HapMap or other sources, we estimated the contribution from Native American ancestry by subtracting the contribution of the European and African ancestries from the observed allele frequencies. The predicted MAF under an admixed model was equal to the sum of contribution from the three ancestries. Pearson's chi-square statistic was used to test Hardy-Weinberg Equilibrium (HWE) for each SNP and to calculate the differences in genotype and allele distributions between Puerto Rican and NHW populations and between the observed and the predicted MAF in Puerto Ricans under an admixture model. Mann-Whitney and Wilcoxon non-parametric tests were used to evaluate statistical differences in MAF and F_ST _means between populations and by SNP function. Frequencies and HWE were corroborated on PowerMarker software [37] and no discrepancies were found with the original results. PowerMarker was used to obtain population-specific and between-population F_ST _values (based on the variance of allele frequencies about the means for the population or between populations) for Puerto Rican and NHW populations; the procedure for such measures was formulated in Weir and Hill [38]. Negative F_ST _values were designated as zero, as they cannot be interpreted biologically [39]. Tests between Puerto Rican and NHW populations were deemed statistically significant at P < 0.0005, after a Bonferroni correction for multiple comparisons (101 tests); tests at P < 0.0007 were deemed significant for our Puerto Rican sample and the predicted Puerto Rican MAF under an admixture model.

## Results

Genotyping data were available for a total of 969 Puerto Rican subjects and 597 NHW subjects. More than 90% of the available samples were genotyped successfully for all but one SNP (*FABP1 *m2353 in NHW). The two populations were similar in age (mean ± standard deviation = 57.9 ± 7.3 for Puerto Ricans, 57.6 ± 9.2 for NHW), but differed in the distribution of sex, with Puerto Ricans having more female subjects than NHW (72.0% versus 52.6%, respectively). However, females represented the majority of subjects for each group.

For each SNP, the genotype distribution, minor allele frequency, sample size, and p-value from chi-square test for difference in genotype distribution and allele frequency among the two populations are detailed (Additional file 2). Frequencies did not differ by sex (data not shown). Twelve SNPs in the Puerto Rican group and ten in the NHW group were not in HWE at P < 0.05. After Bonferroni adjustment for multiple tests, two SNPs were out of HWE: *APOC3 *3U386 (rs5128) in Puerto Ricans, and *FABP1 *m2353 (rs3891700) in both populations. As the genotyping error rate was less than 1%, the lack of HWE could be attributed to chance or to small sample size. None of the SNPs were removed from subsequent analysis.

Of the 101 SNPs, 46 (45.5%) showed allele frequency distributions that were significantly different between the two populations at the P < 0.0005 level. Genotype frequencies were significantly different for 41 SNPs. When comparing the observed Puerto Rican MAF versus the predicted MAF under an admixed model, 24 of the 73 (32.9%) SNPs with available data showed significantly different allele frequencies (Additional file 3). Table 1 shows the mean (standard deviation) MAF for Puerto Ricans and NHW by SNP function. There were no statistically significant differences in mean MAF between populations by SNP function. However, for both populations, non-synonymous SNPs had significantly lower MAF than intronic, promoter or synonymous SNPs. Secondary analysis stratifying by chromosome showed that the SNPs in genes mapping to chromosome 8 had significantly lower mean MAF than most chromosomes in both populations, and that for Puerto Ricans, SNPs in chromosome 3 had lower mean MAF than chromosome 2; Puerto Ricans had significantly higher MAF and lower F_ST _for SNPs in chromosome 4 than NHW (data not shown).

**Table 1:** Mean, standard deviation (SD), and range for MAF and F_ST _by SNP function

**SNP function**	**Number of SNPs**	**Puerto Rican**			**non-Hispanic white**		
		Mean (SD) MAF	Mean (SD) F_ST_	Range F_ST_	Mean (SD) MAF	Mean (SD) F_ST_	Range F_ST_

3'UTR	5	0.20 (0.10)	0.022 (0.050)	0.112	0.19 (0.14)	0.050 (0.067)	0.141

Intron	37	0.31 (0.13)	0.019 (0.029)	0.131	0.29 (0.13)	0.019 (0.032)	0.134

Non-Synonymous	27	0.15 (0.10)^1^	0.041 (0.061)	0.255	0.16 (0.12)^2^	0.033 (0.053)	0.166

Promoter	22	0.30 (0.15)	0.022 (0.061)	0.290	0.27 (0.15)	0.033 (0.058)	0.250

Synonymous	8	0.28 (0.14)	0.036 (0.061)	0.177	0.32 (0.18)	0.013 (0.029)	0.085

^1 ^Significantly different from Puerto Rican intron (p < 0.001), promoter (p = 0.001), synonymous SNPs (p = 0.015)

^2 ^Significantly different from NHW intron (p < 0.001), promoter (p = 0.014), synonymous SNPs (p = 0.027)

**Figure 1:**
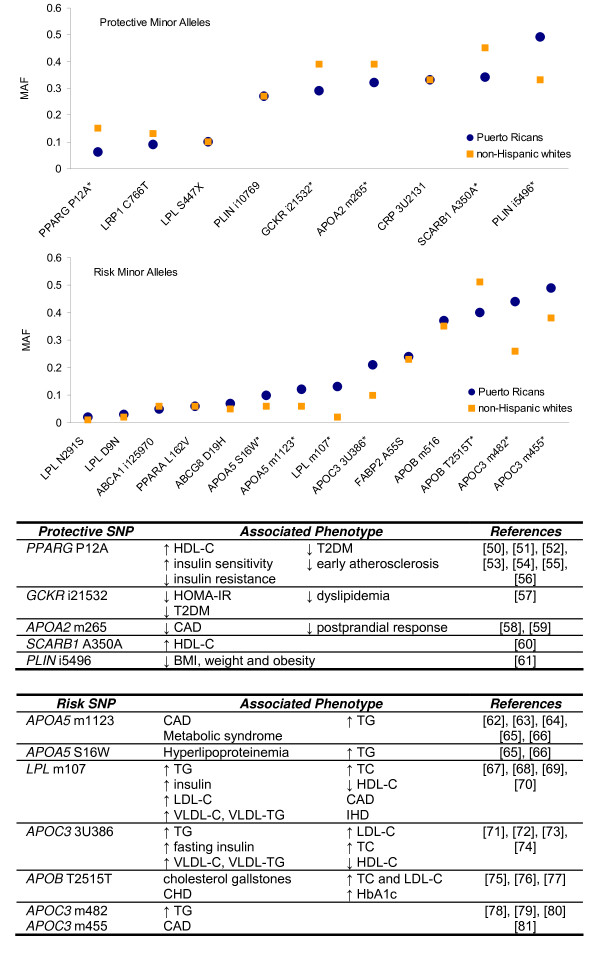
**Differences in protective (upper panel) or risk (lower panel) minor allele frequency in Puerto Rican versus non-Hispanic whites, by SNP**. Tables describe the associated phenotypes for those SNPs with significantly different MAF between populations. *Significantly different MAF between populations, p < 0.0005 Abbreviations: MAF: minor allele frequency; HDL-C: high density lipoprotein; T2DM: type 2 diabetes mellitus; HOMA-IR: homeostasis model assessment of insulin resistance; CAD: coronary artery disease; BMI: body mass index; TG: triglycerides; LDL-C: low density lipoprotein; VLDL-C: very low density lipoprotein; TC: total cholesterol; IHD: ischemic heart disease; CHD: coronary heart disease; HbA1c: glycosylated hemoglobin.

To determine if MAF were over- or under-represented on disease-associated SNPs, alleles were categorized as being either protective or risk based on previously reported associations with prevalence or progression of disease. Figure 1 shows the MAF plotted for each population for those alleles classified as either protective or risk, as well as a description of the phenotypes associated with only those SNPs for which MAF is significantly different between populations at the P < 0.0005 level. Puerto Ricans showed significantly lower frequency of the protective minor allele than NHW in four of the nine SNPs examined, including *PPARG *P12A, *GCKR *i21532, *APOA2 *m265 and *SCARB1 *A350A. These SNPs have been associated with increased high density lipoprotein (HDL-C) and reduced risk or prevalence of type 2 diabetes and coronary disease. Only one protective allele was significantly more frequent in Puerto Ricans, *PLIN *i5496, which has been associated with lower BMI. The remaining four SNPs did not have significantly different allele distributions between the two populations. For the risk minor alleles, Puerto Ricans showed significantly greater frequency in six of the fourteen examined SNPs. Mainly present in genes of lipid metabolic pathways (*APOA5*, *APOB*, *APOC3 *and *LPL*), the higher frequency of risk alleles in Puerto Ricans has been associated previously in other populations with unfavorable lipid profile and insulin response, cardiovascular conditions and metabolic syndrome. Only one SNP had significantly lower frequency of the risk allele in Puerto Ricans than NHW, *APOB *T2515T. The remaining seven SNPs did not have significantly different MAF between populations. Analysis of the 78 SNPs alleles with no conclusive evidence of risk or protection to disease, did not present any differential pattern in MAF distribution between the two populations (data not shown).

Additional file 3 lists the F_ST _values for both populations. The population-specific F_ST _was 0.008 for Puerto Rican and 0.039 for NHW, with a between-population F_ST _of 0.020. The between-population F_ST _suggests that the two populations have little differentiation for the markers tested. Population-specific F_ST _has been suggested as the best indicator of selection over between-population F_ST _[30]; thus, we examined the population-specific F_ST _of each SNP for signals of selection. Table 1 shows the mean (standard deviation) and range for F_ST _for Puerto Ricans and NHW by SNP function. There were no statistical differences in F_ST _between populations or by SNP function.

**Figure 2:**
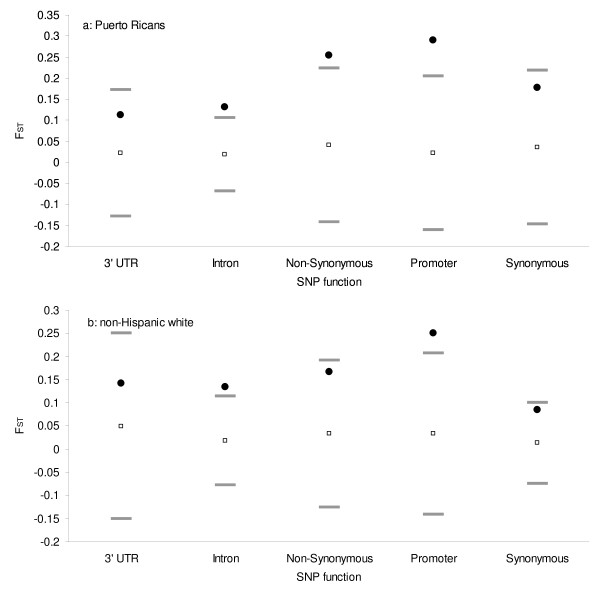
**F_ST _range (filled circle) for Puerto Ricans (a) and non-Hispanic whites (b) by SNP function**. Open squares represent mean F_ST _and bars represent three standard deviations from the mean for each SNP function.

Weir *et al*. have suggested that when the range of the population-specific value exceeds three standard deviations from the mean, such values can be regarded as exceptionally large and may indicate regions under selection [30]. To identify such exceptional values, Figure 2 shows the F_ST _range (filled circles) for Puerto Ricans (a) and NHW (b) by SNP function, with open squares representing mean F_ST _and bars indicating three standard deviations from the mean for each SNP function category. Exceptionally high values in Puerto Ricans were observed in SNPs located in intronic, non-synonymous and promoter regions, while for NHW they were observed in intronic and promoter SNPs. When stratifying by chromosome during secondary analysis, exceptional F_ST _values were positioned in chromosome 11 for Puerto Ricans, while NHW did not show any exceptional values (data not shown).

## Discussion

The present study showed that a group of older adult Puerto Ricans living in the United States had significantly different allele frequency distribution in 101 single nucleotide polymorphisms than similarly aged NHW. Moreover, Puerto Ricans had lower frequency of protective alleles and greater prevalence of risk alleles for SNPs associated with several chronic diseases. Although population differentiation values (F_ST_) did not differ between the two populations, the patterns of F_ST _differed by SNP function in the populations. Puerto Ricans showed exceptional F_ST _values in intronic, non-synonymous and promoter SNPs, while NHW had exceptional values in intronic and promoter SNPs only. These exceptional F_ST _values suggest that selection may be present in genes harboring those SNPs [30].

The differences in allele frequency and population differentiation between the two studied populations may seem surprising as Puerto Ricans comprise an admixed population with European, African and Native American (Taíno) heritage [31]. The value of 45.5% of SNPs showing difference in MAF between the two populations reported here is higher than such differences reported in other populations possibly more distant than Puerto Ricans and NHW. Bamshad *et al*. found that 41% of SNPs differed significantly between African Americans and European Americans [40]. Moreover, Burchard *et al*. reported that studies examining the allele frequency distribution among racial groups had found differences of 15% to 20% between groups [41]. Those authors argue that differences in frequency among ethnic groups are typically not as large as those we observed. It is possible that the origins of the white component of the two populations studied here contribute to some of the observed allele frequency differences. The NHW population shares ancestry primarily with individuals from northern and western Europe, while the Puerto Rican population shares ancestry primarily with peoples of the Iberian Peninsula. To determine the contribution of ancestral populations, we calculated the predicted MAF for Puerto Ricans under an admixture model. Close to a third of the SNPs with available data on predicted MAF showed significantly different allele frequencies from our Puerto Rican sample, suggesting that for those loci, the frequencies cannot be explained by admixture alone. However, for the majority of the analyzed SNPs showing similar observed and expected MAF, each ancestral population may be contributing unequal allele frequencies, supporting our conclusion on the large difference between our Puerto Rican sample and the NHW population. The observation that the MAF in Puerto Ricans tended to be lower for protective alleles and higher for risk alleles in disease-associated SNPs is notable. This could be an important factor in the disparities in chronic diseases observed in Puerto Ricans.

Within genomic regions, other groups have found higher MAF in non-coding regions than in coding regions, reflecting the deleterious effect of mutations in coding regions, with promoter regions having the highest MAF [17]. Our observations agree with these previous results, where non-synonymous SNPs have lower mean MAF than SNPs with other functions. We obtained very low F_ST _values for both populations, suggesting very little population differentiation. To our knowledge, this is the first report of population differentiation for Puerto Ricans. F_ST _estimates for other racial groups have been reported in a wide range from 0.009 in Ashkenazi Jews and HapMap European populations [27] to 0.11 for SNPs across four HapMap populations [29]. The variations are most likely due to differences in sample size and tested markers.

When assessing exceptional F_ST _values by functional categories of the SNP, our study shows that Puerto Ricans had exceptional F_ST _values in intronic, non-synonymous and promoter SNPs. Analysis of data from NHW exhibited a preponderance of exceptional F_ST _values in SNPs mapping to intronic and promoter regions. In secondary analysis, Puerto Ricans showed a prevalence of exceptional F_ST _SNPs mapping to chromosome 11. Interestingly, chromosome 11 includes four clustered apolipoprotein genes (*APOA1*, *APOA4*, *APOA5 *and *APOC3*), for which several gene-environment interactions have been reported. In a study with a Puerto Rican sample, Tang *et al*. showed an excess of Native American ancestry in chromosome 11, which also harbors an olfactory gene cluster that is a target of ongoing positive selection [25]. Kullo and Ding showed high F_ST _values for SNPs in genes of the blood circulation and gas exchange pathways as well as the lipoprotein metabolism pathway in African versus NHW or Asian populations [26]. These observations agree with the prospect that SNPs with high or exceptional F_ST _values should be considered priority candidates for studies on association with complex diseases and local adaptation to environmental influences. Future studies in these populations should focus on intronic, promoter and non-synonymous SNPs for Puerto Ricans, and promoter and intronic SNPs for NHW.

There are limitations to this study. First, the tested SNPs were not randomly selected and thus the frequencies and F_ST _measures may not represent actual distributions, as selective pressures may cause over- or under-representation in disease-associated genes. However, it has been shown that disease-associated SNPs do not show greater population differentiation than SNPs chosen at random [42]. Here, a limited number of tested markers yielded results that could be due to a particular selection. Additionally, statistical power might be limited when stratifying by SNP function (or by chromosome in secondary analysis). Nonetheless, other studies have reported similar measures using fewer SNPs [23,24]. Furthermore, F_ST _values can also indicate genetic drift and bottleneck effect of the populations. Conclusions about selection based on single marker F_ST _values should be interpreted with caution [24]. Still, the F_ST _estimates provide a practical portrayal of the adaptation processes of Puerto Ricans and surely merit further research. Alternatively, genome-wide genotyping using microarrays will allow detection of selection signal at a genome scale based on an excess of ancestral divergence between disease and non-disease groups [25]. It will also be useful to increase the list of tested markers and of biological pathways, as well as to expand into other genetic measures such as linkage disequilibrium in this Puerto Rican cohort. Finally, we defined risk (or protective) allele based on studies on other, often non-Hispanic, populations assuming these alleles would exert the same impact in Puerto Ricans. The amount of risk or protection conferred by a given allele or the extent of change in a biomarker of disease status may be different in Puerto Ricans, and further association studies should be done to corroborate this.

Alternatively, our study has several strengths. First, noting that there are fluctuations in allele and genotype frequencies in control populations by age and sex [43], we sought to compare the two populations using data from individuals within the same age range. We did not observe any differences by sex. Second, our study has a fairly large sample size. Taioli *et al*. showed that the deviation of the estimate of allele frequency calculation from the true frequency decreases with increasing sample size, with sample sizes above 500 having almost no deviation [15]. The same principle applies to F_ST _estimates. Third, Akey *et al*. demonstrated that the average F_ST _values could be affected by genotyping error rates as low as 2–5% [39], but our methods, validated repeatedly, produce less than a 1% genotyping error rate, greatly decreasing the impact of such errors in the F_ST _estimates.

The results of this study have implications in the generation of new hypotheses and selection of candidate genes for association studies and gene-environment interaction studies. Allele frequency variations and F_ST _patterns guide the identification of markers that may have been more prone to local adaptation by past environmental influences that contrast sharply with the environmental pressures faced by Puerto Ricans living in Boston, MA at the present time, namely poor nutritional habits [32]. Such markers are more likely to respond to environmental signals in a manner that manifests as an association with disease status or progression. The observed differences in allele frequency and population differentiation for disease-associated SNPs may explain the disparities in disease prevalence observed in Puerto Ricans. For example, it has been shown that carriers of the variant allele of the Pro12Ala polymorphism in the *PPARG *gene have a beneficial effect on the measures of glucose metabolism with higher fish intake [44]. Taínos (the Amerindian heritage of modern Puerto Ricans) consumed a diet based on fish, tropical fruits and vegetables, and some small animals [45]. Today, major contributors to the Puerto Rican diet are rice, starchy roots, milk, fried meat products, and processed, Westernized foods, with little consumption of fish [46,47]. Interestingly, Puerto Ricans from our cohort had significantly higher frequency of this minor allele than NHW; yet the beneficial effect may have been lost with the low consumption of fish in this population today. This warrants assessment of the contribution of this, and other, polymorphisms to the high prevalence of diabetes in Puerto Ricans [4,48].

Clearly, the goal is to assess genetic and/or environmental contribution to disease to help solve these disparities with targeted interventions and not to create any bias against the populations under study based on genetic profile. Several examples of such ethnicity-based prospective interventions and drug therapies have been reported, such as the population-specific drug-metabolizing capabilities due to various polymorphisms in the cytochrome P450 (CYP450) enzymes [49]. Finally, this study does not define Puerto Ricans on a genetic level, as they are already a well-defined ethnic group with distinct cultural and social norms and environmental influences, even while sharing some characteristics with other Hispanic subgroups. Further studies on genetic and environmental determinants of disease by ethnic or racial groups should recognize the unique genetic and cultural traits of Puerto Ricans.

## Conclusion

Puerto Ricans have significantly different genotype and allele frequency distributions than those of NHW for SNPs in lipid and glucose metabolism, and inflammation response. Puerto Ricans tend to carry risk minor alleles at higher frequency and protective minor alleles at lower frequency than the NHW population. Additionally, patterns of population differentiation were different between the two populations by SNP function. Characterizing the genetic profile of Puerto Ricans will give a solid foundation to the role of genetic variation in disease in an understudied population, support research of genetic interactions with environmental contributors, and help craft interventions to alleviate health disparities in Puerto Ricans.

## Authors' contributions

JM carried out the conception and design of the study, performed the bioinformatics and statistical analysis, interpreted the results, and prepared the draft of the manuscript. LDP assisted in the conception of the study, carried out bioinformatics analysis, and helped draft the manuscript. CQL and JS were involved in data analysis and interpretation, and reviewed the draft of the manuscript. BGB and XA carried out the genotyping and participated in reviewing the draft of the manuscript. DA, SD, KLT and JMO participated in the study design and coordination, data interpretation, and in the revision of the draft the manuscript. All authors read and approved the final manuscript.

## Supplementary Material

Additional file 1 **Gene and genotyping information for 101 SNPs genotyped in Puerto Rican and non-Hispanic white subjects**. Gene variant name, location, function, reference number and the corresponding genotyping primer, probe or assay-on-demand ID for 101 SNPs genotyped in Puerto Rican and non-Hispanic white subjects. Click here for file

Additional file 2 **Allele and genotype frequencies for 101 SNPs in Puerto Ricans and non-Hispanic whites**. Click here for file

Additional file 3 **Predicted minor allele frequencies, and measures of population differentiation for Puerto Ricans and non-Hispanic whites**. Predicted minor allele frequencies under an admixture model, and measures of population-specific and between-population differentiation for Puerto Ricans and non-Hispanic whites. Click here for file
