# Supplementary material for: Disparities in allele frequencies and population differentiation for 101 disease-associated single nucleotide polymorphisms between Puerto Ricans and non-Hispanic whites
Source: BMC Genet. 2009 Aug 14;10:45. doi: 10.1186/1471-2156-10-45 (PMC2734553; doi:10.1186/1471-2156-10-45)
Supplement: Additional file 1 — Gene and genotyping information for 101 SNPs genotyped in Puerto Rican and non-Hispanic white subjects. Gene variant name, location, function, reference number and the corresponding genotyping primer, probe or assay-on-demand ID for 101 SNPs genotyped in Puerto Rican and non-Hispanic white subjects. [file 1471-2156-10-45-S1.doc]

Additional file 1: **Gene and genotyping information for 101 SNPs genotyped in Puerto Rican and non-Hispanic white subjects.**

Description: Gene variant name, location, function, reference number and the corresponding genotyping primer, probe or assay-on-demand ID for 101 SNPs genotyped in Puerto Rican and non-Hispanic white subjects.

| **Gene Symbol and Name** | **Gene Locus** | **SNP name*** | **rs number** | **SNP function** | **Primer and probe or ABI assay-on-demand ID** |
| --- | --- | --- | --- | --- | --- |
| *ABCA1*  ATP-Binding Cassette, Subfamily A, Member 1 | 9q22-q31 | K219R | rs2230806 | Exon 7 | C___2741051_1_ |
|  |  | i125970 | rs2297404 | Intron 33 | C__16186584_10 |
|  |  | i48168 | rs4149272 | Intron 5 | C___2741036_1_ |
|  |  | i27943 | rs2575875 | Intron 2 | C___2741008_1_ |
|  |  | 3U8995 | rs363717 | Exon 50  (3' UTR) | C__11720773_1_ |
| *ABCG5*  ATP-Binding Cassette, Subfamily G, Member 5 | 2p21 | i18429 | rs4148189 | Intron 10 | C__30437456_10 |
|  |  | m216 | rs3806471 | Promoter | C__26135644_10 |
|  |  | Q604E | rs6720173 | Exon 14 | F: CAGCTCAAATGTTTCTGTGACAACT  R: CTGGGCAGGTTTTCTCAATGAATTG  Probe: CCTTCACTCAAGGAATT |
|  |  | i7892 | rs4131229 | Intron 4 | C__27530462_10 |
| *ABCG8*  ATP-Binding Cassette, Subfamily G, Member 8 | 2p21 | C54Y | rs4148211 | Exon 2 | F: AACACCCTGGAGGTCAGAGA  R: CTGCTCCTCTCCCCTTGAAC  Probe: CCTCTACCTGGTAGTTGAG |
|  |  | T400K | rs4148217 | Exon 8 | F: CTCCCGAGTCCTACGAAGATG  R: GGGCGGGTTCAGTAATAAAATGACA  Probe: ATCAGCGTCTTAAACTG |
|  |  | D19H | rs11887534 | Exon 1 | C__26135643_10 |
|  |  | i14222 | rs6709904 | Intron 6 | F: GGGAGGGCATGGAATTCAAGAG  R: GGGCATGTTTTAAGATCTGGTGACT  Probe: TTGTTTCCCTACTGAGTCTG |
| *APOA1*  Apolipoprotein A-I | 11q23 | m3012 | rs11216158 | Promoter | F: TGTATTTTTCCATCAGCTCTGTCCAG_R: TTGCAAGGTATTTGGCTTAAACTAGTCTAA  Probe: AAAGGAAGGAGGTCTTT |
|  |  | m75 | rs670 | Promoter | F: CAGGACCAGTGAGCAGCAA  R: GGCTGGGAGGCTGATAAGC  Probe: CAGCCCCGGCCCT |
|  |  | m2803 | rs2727784 | Promoter | F: CAGCACCCCCTATCCTGATG  R: TTTGAGGGTGGCCTTGCA  Probe: ACTGCAAAGGCTGCA |
|  |  | m2630 | rs613808 | Promoter | F: CACAGGCAAAAATCACAAGGGTAAA  R: GTGGCCAAAGCACTTTCACAA  Probe: CTTAGAATATCCCTATAAGGCT |
| *APOA2*  Apolipoprotein A-II | 1q21-q23 | m265 | rs5082 | Promoter | F: CCAGAGAAATAACTTGGAATCCTGCTT  R: GGTACAGCTCTTAGTGGAGGCTAA  Probe: CTTGGACTTGAATGCAACA |
| *APOA4*  Apolipoprotein A-IV | 11q23 | A4-A5 intergenic | rs1263177 | Intergenic, 3’ near gene | F: GTGGCCTGCCAGTTTGG  R: CTGGCTGTGCTGATGAGACT  Probe: CTGCCCCTAACCCCAGT |
|  |  | N147S | rs5104 | Exon 3 | C__11482766_10 |
|  |  | S367T | rs675 | Exon 3 | F: GGCAGCTCAGCTCTCCAAA  R: CAGCACCTTCAAGGAGAAAGAGA  Probe: CAGGACAAGACTCTCT |
|  |  | T29T | rs5092 | Exon 2 | C___2679569_10 |
|  |  | Q380H | rs5110 | Exon 3 | F: GGCAGCTCAGCTCTCCAAA  R: CCCTGAGCTGGAGCAACA  Probe: CTGCTCCTGCTGCTG |
|  |  | m35 | rs5090 | Promoter | F: TGGAGTCACACTGAGGAAGGA  R: TGGGAACTGACTGAAGCTCAGA  Probe: AGACATTTAAACTCTCTCCC |
| *APOA5*  Apolipoprotein A-V | 11q23 | m1123 | rs662799 | Promoter | F: CCCTGCGAGTGGAGTTCA  R: CTCTGAGCCCCAGGAACTG  Probe: CGAAAGTAAGATTTGCC |
|  |  | S16W | rs3135506 | Exon 2 | F: CCAGGCCCTGATTACCTAGTC  R: GAAGTAGTCCCAGAAGCCTTTCC  Probe: CAGCGTTTTCGGCCAC |
| *APOB*  Apolipoprotein B | 2p24 | A618V | rs679899 | Exon 14 | C___1026583_10 |
|  |  | m516 | rs934197 | Promoter | F: TCACCAGACCTCCCTGCAT  R: GGATTTGCACCCACACCCTA  Probe: CTCTCTTCTCCCCCAGCACGG |
|  |  | E4181K | rs1042031 | Exon 29 | F: TGTTTGATGGCTTGGTACGAGTTA  R: ATCAATGAGTGAGTCAATCAGATGCTT  Probe: ACTTTCATATGGAATTCTTGAG |
|  |  | P2739L | rs676210 | Exon 26 | C___3216558_10 |
|  |  | T2515T | rs693 | Exon 26 | F: GGCTCACATGAAGGCCAAATTC  R: AGTTCCTGCTGAATGTCCATTTGAT  Probe: TCTTCTAGGGTCTCTCG |
| *APOC3*  Apolipoprotein C-III | 11q23 | G34G | rs4520 | Exon 3 | C___1073222_1_ |
|  |  | 3U386 | rs5128 Exon 4 (3’UTR) | | C___8907537_1_ |
|  |  | m2886 | rs2542051 | Promoter | F: GGTCAGTCCAGAGGTCAGAGT  R: GAGGCACATGTCCGTGTGA  Probe: ATCTGAGTTTTCTGCTCCT |
|  |  | m640 | rs2542052 | Promoter | F: CGGCCTTGGCCCTTCTC  R: GCCCCCACCCTGTGT  Probe: CCTCCCCCTTAGTGTAG |
|  |  | m455 | rs2854116 | Promoter | F: TGCCGGAGCCACTGATG  R: CTGAACACAGCCTGGAGTAGAG  Probe: TTTACTCCAAACACCCCCCA |
|  |  | m482 | rs2854117 | Promoter | F: AGAGCTCAGCCCTGTAACCA  R: GGGCTTCTTCAGACTTGAGAACAA  Probe: CACAGAAGACCAGGCAT |
| *APOE*  Apolipoprotein E | 19q13.2 | m226 | rs405509 | Promoter | F: CTTGGCCCCCAGAATGGA  R: CCCCACAGTCCCCAGGAA  Probe: CCCAGTAATACAGACACC |
|  |  | R176C | rs7412 | Exon 4 | C____904973_10 |
|  |  | C130R | rs429358 | Exon 4 | C___3084793_20 |
| *ATF6*  Activating Transcription Factor 6 | 1q22-q23 | i190554 | rs2499856 | Intron 15 | C__16191886_10 |
| *CRP*  C-Reactive Protein | 1q21-q23 | 3U2131 | rs1205 | Exon 2 (3'UTR) | C___7479334_10 |
|  |  | i178 | rs1417938 | Intron 1 | C___7479322_10 |
| *CYP7A1*  Cytochrome P450, Subfamily VIIA, Polypeptide 1 | 8q11-q12 | i6782 | rs11786580 | Intron 4 | C___3051547_10 |
|  |  | Intergenic 3U12536 | rs10957056 | Intergenic | C___3051550_10 |
| *FABP1*  Fatty Acid-Binding Protein 1 | 2p11 | T94A | rs2241883 | Exon 3 | C__25473098_10 |
|  |  | m2353 | rs3891700 | Promoter | F: TGCCATCCTGGAGCTCTTCT  R: GGAGAGAGGCCACCAAGAG  Probe: AGTGACCGCAAGGCA |
| *FABP2*  Fatty Acid-Binding Protein 2 | 4q28-q31 | A55S | rs1799883 | Exon 2 | C____761961_10 |
|  |  | m193 | rs6857641 | Promoter | F: GACGGCATGCATTCTAATTCTTGAA  R: CGTTCTGTACATTCCTGAGATCTTCTG  Probe: CACAAACTGTAATTTAAG |
|  |  | m767 | rs10034661 | Promoter | F: GCCTGAAGTAGGGAAAGGAGAGAT  R: GAGTCAGAAGCCACAGCTCAATTAT  Probe: CCCTGACCCCACATAC |
| *GCKR*  Glucokinase Regulatory Protein | 2p23.3-p23.2 | i21532 | rs780094 | Intron 16 | C___2862873_10 |
| *LIPC*  Hepatic Lipase | 15q21-q23 | i33753 | rs7169744 | Intron 1 | C__32075190_10 |
|  |  | V95M | rs6078 | Exon 3 | F: TGCGACCCTCCCTCTGT  R: AGCGCGGCCACCAT  Probe: TGGACGGCATGCTAG |
|  |  | T224T | rs6084 | Exon 5 | C____305150_10 |
|  |  | i618 | rs8034802 | Intron 1 | C___1929300_10 |
|  |  | i67180 | rs1973028 | Intron 1 | C__11668958_10 |
| *LIPG*  Endothelial Lipase | 19q21.1 | i13576 | rs2276269 | Intron 5 | C__25473466_10 |
|  |  | i24582 | rs6507931 | Intron 8 | C___1519436_10 |
|  |  | T111I | rs2000813 | Exon 3 | F: GCTGCACAAACTCGTGTCA  R: CCAGTCAACCACAACTACATTGG  Probe: CGTCTTTCTCTCTTGTGTGCA |
| *LPL*  Lipoprotein Lipase | 8p22  8p22 | D9N (D36N) | rs1801177 | Exon 2 | F: CCCTCCAGTTAACCTCATATCCAATTTT  R: GTGTCCTCAGCTGTGTCTTCAG  Probe: TTTCGATGTCGATAAAA |
|  |  | N291S (N318S) | rs268 | Exon 6 | F: AAAGAACCGCTGCAACAATCTG  R: AGGTACATTTTGCTGCTTCTTTTGG  Probe: CTATGAGATCAATAAAGTC |
|  |  | S447X (S474X) | rs328 | Exon 9 | C____901792_1_ |
|  |  | m107 (m93) | rs1800590 | Promoter | F: GCAAATAAACCTCATCACCTATTGGCTAT  R: GGGTTGATCCTCATTACTGTTTGCT  Probe: TGAATTTAGGTCCCTCCC |
| *LRP1*  Low Density Lipoprotein Receptor-Related Protein 1 | 12q13.1-q13.3 | i10701 | rs715948 | Intron 2 | C___1955080_10 |
|  |  | C766T | rs1799986 | Exon 3 | C___1955081_10 |
|  |  | i68477 | rs1800191 | Intron 55 | F: GCCCAGACCCTGCCA  R: AGCCAGGGCGACAGC  Probe: CACTGCAGGCACAGG |
| *MTTP*  Microsomal Triglyceride Transfer Protein | 4q22-q24 | C174C | rs982424 | Exon 5 | C___1192256_1_ |
|  |  | i10249 | rs1800591 | Intron 1 | C___8934089_10 |
|  |  | i9314 | rs3811800 | Intron 1 | F: GCACTTTCACATTGCCTTGTATTCA  R: CGAAGTTTTCCGGGAGTTAAACAGA  Probe: CTTTGTTAAAGAAATATAAGTTT |
| *NOS3*  Nitric Oxide Synthase 3 | 7q36 | m459 | rs11771443 | Promoter | F: TGCAGAGGAGGGAAGAAGAAGAA  R: GCGTCTGTGGGCGTAACAT  Probe: CTGATGTGAGGCCC |
|  |  | i19342 | rs743507 | Intron 21 | C___1026883_20 |
|  |  | i1103 | rs1800783 | Intron 1 | C___3219463_1_ |
|  |  | E298D | rs1799983 | Exon 8 | F: GGCTGGACCCCAGGAAA  R: CACCCAGTCAATCCCTTTGGT  Probe: CCCAGATGAGCCCCCA |
| *PDZK1*  PDZ Domain-Containing 1 | 1q21 | i19738 | rs1284300 | Intron 3 | C___1862256_10 |
| *PLIN*  Perilipin | 15q26 | 3U2197 (PLIN6) | rs1052700 | Exon 9 | F: TGATCTGTTCCCCCTCTGATGAA  R: CACCCAAGAGCTTTTGCATCTG /  Probe: CTGATGATCAAGGCTCC |
|  |  | i10769 (PLIN4) | rs894160 | Intron 6 | C___8722593_10  F: CTGGTATCTCCTGAGGCACATT  R: CATCCCCAAAAGCTGTGTAATAAGG  Probe: CTCCCTAGAGGTTTAG |
|  |  | i5496 (PLIN1) | rs2289487 | Intron 2 | C__15881785_20 |
|  |  | P371P (PLIN5) | rs2304795 | Exon 8 | F: GCCCTCCCCTTGGTTGAG  R: AGGGAGGGTGCTGCAC  Probe: CACCAGCCCCTGCTGT |
| *PPARA*  Peroxisome Proliferator-Activated Receptor-Alpha | 22q12-q13.1 | L162V | rs1800206 | Exon 5 | F: CAGAAACAAATGCCAGTATTGTCGAT  R: CCTTACCTACCGTTGTGTGACATC  Probe: ACAAGTGCCTTTCTG |
|  |  | i5522 | rs135549 | Intron 2 | C___2988819_1_ |
| *PPARG*  Peroxisome Proliferator-Activated Receptor-Gamma  (referring to mRNA NM_015869.4) | 3p25  3p25 | H477H | rs3856806 | Exon 7 | F: CTCCAGAAAATGACAGACCTCAGA  R: GTCTGTCTCCGTCTTCTTGATCAC  Probe: CTGCACGTGTTCCG |
|  |  | m39803 | rs10865710 | Promoter | F: CCTGATGATAAGGCTTTTGGCATT  R: TCTTATGAAAGGCTCAAGGATCCTAGA  Probe: CTGTATTTTCCATGAAGACA |
|  |  | m2866 | rs12497191 | Promoter | F: ATACCCTTCTGTCTCCAAAGTCCTA  R: CTGTTGAGGGATGTAAGGTTCTGT  Probe: TCAACCTCTGCATTGC |
|  |  | P12A | rs1801282 | Exon 1 | C___1129864_10 |
| *PPARGC1A*  Peroxisome Proliferator-Activated Receptor-Gamma, Coactivator 1, Alpha | 4p15.1 | i27289 | rs4235308 | Intron 2 | F: GAAGTCATGGAGTGCTTGATAAGGA  R: CTGGCGAATTATCAAACTCAAAGCA  Probe: TTTGCCCCACATAGTAA |
|  |  | i5378 | rs2946385 | Intron 2 | C___1643232_10 |
|  |  | m1668 | rs2970869 | Promoter | F: CATTCCCGAGGTTGTATTTTCCT  R: ATACAGTTCACCAGCACATTTTCC  Probe: TCTGCTGTTTATAAAAG |
|  |  | T612M | rs3736265 | Exon 9 | F: GTTAAGTGGCAGTTGCAAATGC  R: GTAGGGCGATCTTGAACGTGAT  Probe: CGGTGCGTGCGGTGT |
|  |  | i55301 | rs4697046 | Intron 2 | F: ACAGTTAAATAACCTCAATTTAATTTTTGTATGTGGG  R: GATATTCCTTATTTTCTGCCCAGGATCA  Probe: ATTGAAATCTTTGAATACCAG |
|  |  | 3U4898 | rs3774923 | 3'UTR | F: CGCCCAACACGATCTTGTAAGA  R: GCATCCGACAGGACAAACAGT  Probe: TTGTGTTCTGAATGAATC |
| *SCARB1*  Scavenger Receptor Class B, Member 1 | 12q24.31 | A350A | rs5888 | Exon 8 | C___7497008_1_ |
|  |  | i9107 | rs4765181 | Intron 1 | F: TGCCCGGGCCAACAT  R: GGAATCTGGGTATATTTAGATGATGAAGCT  Probe: CACTTCCCGCAGAGAA |
|  |  | G2S | rs4238001 | Exon 1 | C__26062113_10 |
|  |  | i82699 | rs701106 | Intron 12 | F: CCTTCCTGTCCATCTGGGAGTAG  R: GAAGGCCTTGCCCCCAA  Probe: AATATTAACACTCCACCCCAAC |
|  |  | i30026 | rs10846748 | Intron 1 | F: GCGTGGAGCTTCCCATTAAAGA  R: GCAGGCTTCGCTAGAAACAAG  Probe: TCCTGCCGGGTCCA |
|  |  | i19960 | rs3924313 | Intron 1 | F: CATGTGCTTTTCTGAGGTGTGAATT  R: AACATGGCCCTCAAAATCTCTGA  Probe: CCTGAGGACTTATGAAAG |
|  |  | i51973 | rs61932577 | Intron 5 | F: CAAGTGGAACGGGCTGAGCAAGGT  R: TCTGGTCCCTGCCACTCCCGA  Probe: AGCCATGGCCAGGCCCACCC |
| *WDTC1*  WD and Tetratricopeptide Repeats 1 | 1p36.11 | i22835 | rs4460661 | Intron 1 | C__27905442_10 |
|  |  | i61970 | rs3813790 | Intron 10 | C__11597741_10 |
| *ZNF568*  Zinc Finger Protein 568 | 19q13 | i23579 | rs544543 | Intron 6 | C____601962_10 |
|  |  | i23072 | rs505717 | Intron 6 | C____601964_10 |

* Variant name for amino acid change, or intronic (i), promoter (m), or in/near 3’UTR (3U) position from mRNA start.
