# Supplementary material for: Disparities in allele frequencies and population differentiation for 101 disease-associated single nucleotide polymorphisms between Puerto Ricans and non-Hispanic whites
Source: BMC Genet. 2009 Aug 14;10:45. doi: 10.1186/1471-2156-10-45 (PMC2734553; doi:10.1186/1471-2156-10-45)
Supplement: Additional file 2 — Allele and genotype frequencies for 101 SNPs in Puerto Ricans and non-Hispanic whites. [file 1471-2156-10-45-S2.doc]

**Additional file 2 - Allele and genotype frequencies for 101 SNPs in Puerto Ricans and non-Hispanic whites**

| **SNP** | **Puerto Rican** | **NHW** | **p-value*** | **SNP** | **Puerto Rican** | **NHW** | **p-value*** | **SNP** | **Puerto Rican** | **NHW** | **p-value*** |
| --- | --- | --- | --- | --- | --- | --- | --- | --- | --- | --- | --- |
| *ABCA1 K219R (rs22308060)* | | | | *ABCA1 i125970 (rs2297404)* | | | | *ABCA1 i48168 (rs4149272)* ^1^ | | | |
| N  GG  GA  AA  MAF | 947  36.0  46.5  17.5  0.41 | 596  57.7  36.5  5.9  0.24 | < 0.0001  < 0.0001 | N  GG  GC  CC  MAF | 949  89.8  10.2  0  0.05 | 596  88.1  11.2  0.7  0.06 | 0.0326  0.1633 | N  CC  CT  TT  MAF | 956  32.6  44.8  22.6  0.45 | 596  45.3  44.5  10.2  0.32 | < 0.0001  < 0.0001 |
| *ABCA1 i27943 (rs2575875)* | | | | *ABCA1 3U8995 (rs363717)* | | | | *ABCG5 i18429 (rs4148189)* | | | |
| N  GG  GA  AA  MAF | 954  29.8  49.9  20.3  0.45 | 597  43.9  45.2  10.9  0.34 | <0.0001  <0.0001 | N  TT  TC  CC  MAF | 936  62.2  33.8  4.1  0.21 | 596  79.4  18.8  1.8  0.11 | < 0.0001  < 0.0001 | N  CC  CT  TT  MAF | 952  54.2  38.3  7.5  0.27 | 593  79.8  19.1  1.2  0.11 | < 0.0001  < 0.0001 |
| *ABCG5 m216 (rs3806471)* ^1^ | | | | *ABCG5 Q604E (rs6720173)* | | | | *ABCG5 i7892 (rs4131229)* | | | |
| N  TT  TG  GG  MAF | 961  47.7  39.5  12.8  0.33 | 596  42.3  44.1  13.6  0.36 | 0.1119  0.0769 | N  CC  CG  GG  MAF | 965  57.1  36.1  6.8  0.25 | 596  67.1  29.7  3.2  0.18 | < 0.0001  < 0.0001 | N  AA  AG  GG  MAF | 948  47.6  42.5  9.9  0.31 | 596  33.9  46.3  19.8  0.43 | < 0.0001  < 0.0001 |
| *ABCG8 C54Y (rs4148211)* ^2^ | | | | *ABCG8 T400K (rs4148217)* | | | | *ABCG8 D19H (rs11887534)* ^2^ | | | |
| N  TT  TC  CC  MAF | 966  51.1  39.5  9.3  0.29 | 596  37.9  44.1  18.0  0.40 | < 0.0001  < 0.0001 | N  GG  GT  TT  MAF | 958  60.8  34.2  5.0  0.22 | 597  66.3  30.2  3.5  0.19 | 0.0624  0.0181 | N  GG  GC  CC  MAF | 961  87.0  12.6  0.3  0.07 | 597  90.8  8.4  0.8  0.05 | 0.0123  0.0625 |
| *ABCG8 i14222 (rs6709904)* ^2^ | | | | *APOA1 m3012 (rs11216158)* | | | | *APOA1 m75 (rs670)* | | | |
| N  TT  TC  CC  MAF | 949  68.5  27.5  4.0  0.18 | 596  82.6  15.8  1.7  0.10 | < 0.0001  < 0.0001 | N  GG  GA  AA  MAF | 958  70.5  27.3  2.2  0.16 | 596  76.7  21.5  1.8  0.13 | 0.0271  0.0118 | N  CC  CT  TT  MAF | 963  69.4  28.7  2.0  0.16 | 597  70.4  26.5  3.2  0.16 | 0.2342  0.9344 |
| *APOA1 m2803 (rs2727784)* | | | | *APOA1 m2630 (rs613808)* | | | | *APOA2 m265 (rs5082)* | | | |
| N  GG  GA  AA  MAF | 939  28.5  47.7  23.7  0.48 | 596  12.4  48.7  38.9  0.63 | < 0.0001  < 0.0001 | N  GG  GA  AA  MAF | 948  26.1  48.0  25.9  0.50 | 597  47.6  43.7  8.7  0.31 | < 0.0001  < 0.0001 | N  AA  AG  GG  MAF | 957  46.4  43.3  10.3  0.32 | 597  38.0  45.1  16.9  0.39 | < 0.0001  0.0001 |
| *APOA4 A4-A5 Intergenic (rs1263177)* | | | | *APOA4 N147S (rs5104)* | | | | *APOA4 S367T (rs675)* | | | |
| N  TT  TC  CC  MAF | 957  38.2  49.2  12.5  0.37 | 596  38.1  47.7  14.3  0.38 | 0.6026  0.5988 | N  AA  AG  GG  MAF | 959  67.5  29.3  3.2  0.18 | 596  73.3  24.8  1.8  0.14 | 0.0289  0.0082 | N  AA  AT  TT  MAF | 951  72.0  25.1  2.8  0.15 | 597  65.0  31.0  4.0  0.20 | 0.0125  0.0030 |
| *APOA4 T29T (rs5092)* | | | | *APOA4 Q380H (rs5110)* ^2^ | | | | *APOA4 m35 (rs5090)* | | | |
| N  AA  AG  GG  MAF | 940  65.1  31.6  3.3  0.19 | 596  65.3  31.2  3.5  0.19 | 0.9638  0.9826 | N  CC  CA  AA  MAF | 959  89.5  10.5  0  0.05 | 597  81.9  18.1  0  0.09 | < 0.0001  < 0.0001 | N  CC  CG  GG  MAF | 958  95.9  4.1  0  0.02 | 597  89.6  10.2  0.2  0.05 | < 0.0001  < 0.0001 |
| *APOA5 m1123 (rs662799)* | | | | *APOA5 S16W (rs3135506)* | | | | *APOB A618V (rs679899)* ^2^ | | | |
| N  AA  AG  GG  MAF | 962  77.7  20.7  1.7  0.12 | 592  88.9  11.1  0  0.06 | < 0.0001  < 0.0001 | N  CC  CG  GG  MAF | 953  80.2  19.0  0.8  0.10 | 597  89.3  10.1  0.7  0.06 | < 0.0001  < 0.0001 | N  GG  GC  AA  MAF | 965  51.8  39.6  8.6  0.28 | 596  24.8  55.4  19.8  0.47 | < 0.0001  < 0.0001 |
| *APOB m516 (rs934197)* ^1^ | | | | *APOB E4181K (rs1042031)* | | | | *APOB P2739L (rs676210)* | | | |
| N  CC  CT  TT  MAF | 948  43.4  39.6  17.1  0.37 | 597  47.7  47.1  11.2  0.35 | 0.0011  0.2343 | N  CC  CT  TT  MAF | 966  73.7  23.9  2.4  0.14 | 596  68.1  28.5  3.7  0.18 | 0.0400  0.0100 | N  CC  CT  TT  MAF | 955  65.2  31.3  3.5  0.19 | 596  61.2  33.1  5.7  0.22 | 0.0619  0.0355 |
| *APOB T2515T (rs693)* | | | | *APOC3 G34G (rs4520)* | | | | *APOC3 3U386 (rs5128)* ^1^ | | | |
| N  GG  GA  AA  MAF | 953  36.6  47.5  15.8  0.40 | 596  24.3  49.3  26.3  0.51 | < 0.0001  < 0.0001 | N  CC  CT  TT  MAF | 957  48.1  42.5  9.5  0.31 | 597  52.6  40.0  7.4  0.27 | 0.1474  0.0508 | N  CC  CG  GG  MAF | 945  57.1  27.2  15. 7  0.21 | 597  80.1  19.6  0.3  0.10 | < 0.0001  < 0.0001 |
| *APOC3 m2886 (rs2542051)* | | | | *APOC3 m640 (rs2542052)* | | | | *APOC3 m455 (rs2854116)* | | | |
| N  TT  TG  GG  MAF | 959  27.3  52.5  20.2  0.46 | 597  39.0  46.6  14.4  0.38 | < 0.0001  < 0.0001 | N  GG  GT  TT  MAF | 944  26.0  50.6  23.4  0.49 | 597  37.4  48.1  14.6  0.39 | < 0.0001  < 0.0001 | N  TT  TC  CC  MAF | 958  26.5  49.7  23.8  0.49 | 596  37.6  48.3  14.1  0.38 | < 0.0001  < 0.0001 |
| *APOC3 m482 (rs2854117)* | | | | *APOE m226 (rs405509)* | | | | *APOE R176C (rs7412)* | | | |
| N  GG  GA  AA  MAF | 964  32.0  49.0  19.1  0.44 | 596  53.0  41.1  5.9  0.26 | < 0.0001  < 0.0001 | N  CC  CA  AA  MAF | 952  31.9  50.7  17.3  0.43 | 597  26.0  51.1  22.9  0.48 | 0.0056  0.0016 | N  CC  CT  TT  MAF | 948  90.1  9.7  0.2  0.05 | 597  84.6  14.9  0.2  0.08 | 0.0005  0.0037 |
| *APOE C130R (rs429358)* | | | | *ATF6 i190554 (rs2499856)* | | | | *CRP 3U2131 (rs1205)* | | | |
| N  TT  TC  CC  MAF | 954  74.4  23.9  1.7  0.14 | 597  69.2  28.0  2.8  0.17 | 0.0461  0.0145 | N  GG  GA  AA  MAF | 963  84.0  15.0  1.0  0.09 | 590  78.3  19.8  1.9  0.12 | 0.0144  0.0029 | N  CC  CT  TT  MAF | 941  45.1  42.9  12.0  0.33 | 597  46.7  41.0  12.2  0.33 | 0.7598  0.6761 |
| *CRP i178 (rs1417938)* | | | | *CYP7A1 i6782 (rs11786580)* | | | | *CYP7A1 Intergenic 3U12536 (rs10957056)* | | | |
| N  TT  TA  AA  MAF | 960  55.4  38.1  6.5  0.26 | 597  48.6  40.0  11.4  0.31 | 0.0008  0.0004 | N  CC  CT  TT  MAF | 956  68.5  28.7  2.8  0.17 | 596  63.8  32.2  4.0  0.20 | 0.1105  0.0368 | N  AA  AC  CC  MAF | 960  68.8  27.6  3.6  0.17 | 597  65.5  30.0  4.5  0.20 | 0.3643  0.1466 |
| *FABP1 T94A (rs2241883)* ^1, 2^ | | | | *FABP1 m2353 (rs3891700)* ^1, 2^ | | | | *FABP2 A55S (rs1799883)* | | | |
| N  AA  AG  GG  MAF | 969  50.5  43.2  6.3  0.28 | 597  46.9  40.0  13.1  0.33 | < 0.0001  0.0022 | N  GG  GA  AA  MAF | 908  65.4  19.4  15.2  0.25 | 528  72.9  15.3  5.4  0.19 | 0.0131  0.0008 | N  CC  CT  TT  MAF | 952  58.6  35.5  5.9  0.24 | 597  58.6  37.4  4.0  0.23 | 0.2453  0.5478 |
| *FABP2 m193 (rs6857641)* ^1^ | | | | *FABP2 m767 (rs10034661)* | | | | *GCRK i21532 (rs780094)* | | | |
| N  GG  GA  AA  MAF | 962  38.3  43.6  18.2  0.40 | 597  33.5  49.6  16.9  0.42 | 0.0627  0.3363 | N  CC  CT  TT  MAF | 958  55.4  37.8  6.8  0.26 | 597  58.5  37.5  4.0  0.23 | 0.0634  0.0679 | N  CC  CT  TT  MAF | 881  51.0  39.4  9.6  0.29 | 576  36.6  49.5  13.9  0.39 | < 0.0001  < 0.0001 |
| *LIPC i33753 (rs7169744)* | | | | *LIPC V95M (rs6078)* ^1^ | | | | *LIPC T224T (rs6084)* ^2^ | | | |
| N  CC  CG  GG  MAF | 964  78.1  20.4  1.5  0.12 | 596  74.3  23.7  2.0  0.14 | 0.2072  0.0747 | N  GG  GA  AA  MAF | 954  90.8  8.6  0.6  0.05 | 597  95.8  4.0  0.2  0.02 | 0.0009  0.0001 | N  CC  CG  GG  MAF | 942  30.7  47.0  22.3  0.46 | 595  16.8  54.6  28.6  0.56 | < 0.0001  < 0.0001 |
| *LIPC i618 (rs8034802)* | | | | *LIPC i67180 (rs1973028)* | | | | *LIPG i13576 (rs2276269)* | | | |
| N  TT  TA  AA  MAF | 955  45.4  42.2  12.4  0.33 | 597  54.1  38.7  7.2  0.27 | 0.0003  0.0001 | N  TT  TC  CC  MAF | 954  32.5  47.7  19.8  0.44 | 597  40.5  45.7  13.7  0.37 | 0.0006  0.0001 | N  CC  CT  TT  MAF | 962  33.6  47.4  19.0  0.46 | 597  27.3  50.6  22.1  0.47 | 0.0279  0.0106 |
| *LIPG i24582 (rs6507931)* | | | | *LIPG T111I (rs2000813)* | | | | *LPL D9N (rs1801177)* | | | |
| N  CC  CT  TT  MAF | 958  26.5  47.1  26.4  0.50 | 597  22.1  50.8  27.1  0.53 | 0.1373  0.1641 | N  GG  GA  AA  MAF | 965  57.7  36.1  6.2  0.24 | 597  56.3  36.0  7.7  0.26 | 0.5126  0.3575 | N  CC  CT  TT  MAF | 965  94.2  5.6  0.2  0.03 | 597  97.5  2.5  0  0.02 | 0.0083  0.0017 |
| *LPL N291S (rs268)* ^1^ | | | | *LPL S447X (rs328)* | | | | *LPL m107 (rs1800590)* ^1^ | | | |
| N  AA  AG  GG  MAF | 965  96.7  3.1  0.2  0.02 | 596  98.3  1.7  0  0.01 | 0.1176  0.0336 | N  CC  CG  GG  MAF | 955  81.2  17.9  0.9  0.10 | 597  81.4  18.1  0.5  0.10 | 0.6284  0.7509 | N  TT  TG  GG  MAF | 959  77.0  20.2  2.8  0.13 | 597  96.6  3.4  0  0.02 | < 0.0001  < 0.0001 |
| *LRP1 i10701 (rs715948)* | | | | *LRP1 C766T (rs1799986)* | | | | *LRP1 i68477 (rs1800191)* | | | |
| N  CC  CT  TT  MAF | 949  54.8  39.2  6.0  0.26 | 597  49.6  41.4  9.0  0.30 | 0.0297  0.0121 | N  CC  CT  TT  MAF | 948  82.1  16.9  1.1  0.09 | 597  74.9  23.6  1.5  0.13 | 0.0031  0.0009 | N  GG  GA  AA  MAF | 954  37.0  47.2  15.8  0.39 | 597  44.4  46.4  9.2  0.32 | 0.0002  0.0001 |
| *MTTP C174C (rs982424)* | | | | *MTTP i10249 (rs1800591)* ^2^ | | | | *MTTP i9314 (rs3811800)* | | | |
| N  AA  AG  GG  MAF | 952  81.8  16.8  1.4  0.10 | 596  87.2  12.8  0  0.06 | 0.0012  0.0010 | N  CC  CA  CC  MAF | 961  54.1  38.7  7.2  0.27 | 596  63.4  30.5  6.0  0.21 | 0.0014  0.0010 | N  AA  AG  GG  MAF | 951  35.9  47.0  17.1  0.41 | 596  49.8  40.4  9.7  0.30 | < 0.0001  < 0.0001 |
| *NOS3 m459 (rs11771443)* | | | | *NOS3 i19342 (rs743507)* | | | | *NOS3 i1103 (rs1800783)* | | | |
| N  GG  GA  AA  MAF | 958  62.0  33.4  4.6  0.21 | 597  72.0  25.6  2.3  0.15 | 0.0001  < 0.0001 | N  TT  TC  CC  MAF | 958  61.3  33.1  5.6  0.22 | 597  55.9  37.9  6.2  0.25 | 0.1135  0.0590 | N  TT  TA  AA  MAF | 962  36.2  48.2  15.3  0.40 | 597  37.9  47.7  14.4  0.38 | 0.7706  0.4770 |
| *NOS3 E298D (rs1799983)* | | | | *PDZK1 i19738 (rs1284300)* | | | | *PLIN 3U2197 (rs1052700)* | | | |
| N  GG  GT  TT  MAF | 957  57.8  36.1  6.2  0.24 | 597  47.6  42.5  9.9  0.31 | 0.0001  < 0.0001 | N  CC  CT  TT  MAF | 948  85.1  14.5  0.4  0.08 | 597  84.6  14.2  1.2  0.08 | 0.2319  0.5182 | N  TT  TA  AA  MAF | 957  62.1  32.5  5.4  0.22 | 559  41.9  46.2  12.0  0.35 | < 0.0001  < 0.0001 |
| *PLIN i10769 (rs894160)* | | | | *PLIN i5496 (rs2289487)* | | | | *PLIN P371P (rs2304795)* | | | |
| N  GG  GA  AA  MAF | 953  53.7  38.5  7.8  0.27 | 578  54.7  37.4  8.0  0.27 | 0.9051  0.8199 | N  CC  CT  TT  MAF | 947  26.3  48.8  24.9  0.49 | 578  45.7  42.9  11.4  0.33 | < 0.0001  < 0.0001 | N  TT  TC  CC  MAF | 955  38.8  48.0  13.2  0.37 | 577  39.9  48.8  11.8  0.36 | 0.7156  0.5005 |
| *PPARA L162V (rs1800206)* ^2^ | | | | *PPARA i5522 (rs135549)* | | | | *PPARG H477H (rs3856806)* | | | |
| N  CC  CG  GG  MAF | 949  88.7  10.7  0.5  0.06 | 596  88.4  10.6  0. 8  0.06 | 0.7549  0.7990 | N  CC  CT  TT  MAF | 941  25.6  49.0  25.4  0.50 | 596  21.6  48.2  30.2  0.54 | 0.0631  0.0178 | N  GG  GA  AA  MAF | 957  86.0  13.3  0.5  0.07 | 596  74.7  23.7  1.7  0.14 | < 0.0001  < 0.0001 |
| *PPARG m39803 (rs10865710)* | | | | *PPARG m2866 (rs12497191)* | | | | *PPARG P12A (rs1801282)* | | | |
| N  GG  GC  CC  MAF | 953  59.1  35.7  5.2  0.23 | 596  52.7  41.3  6.0  0.27 | 0.0474  0.0236 | N  AA  AG  GG  MAF | 955  77.0  21.9  1.2  0.12 | 596  76.8  21.1  2.0  0.13 | 0.3807  0.6861 | N  CC  CG  GG  MAF | 952  87.4  12.3  0.3  0.06 | 596  72.3  25.5  2.2  0.15 | < 0.0001  < 0.0001 |
| *PPARGC1A i27289 (rs4235308)* | | | | *PPARGC1A i5378 (rs2946385)* ^1^ | | | | *PPARGC1A m1668 (rs2970869)* | | | |
| N  AA  AG  GG  MAF | 962  37.4  47.1  15.5  0.39 | 597  38.7  46.4  14.9  0.38 | 0.8711  0.6058 | N  GG  GT  TT  MAF | 957  34.2  45.2  20.6  0.43 | 597  35.7  48.6  15.7  0.40 | 0.0574  0.0811 | N  GG  GA  AA  MAF | 951  54.6  38.7  6.7  0.26 | 587  60.1  34.8  5.1  0.22 | 0.0799  0.0249 |
| *PPARGC1A T612M (rs3736265)* | | | | *PPARGC1A i55301 (rs4697046)* | | | | *PPARGC1A 3U4898 (rs3774923)* | | | |
| N  GG  GA  AA  MAF | 960  88.0  11.6  0.5  0.06 | 597  89.8  10.2  0  0.05 | 0.2000  0.2055 | N  AA  AG  GG  MAF | 957  37.9  49.2  12.9  0.37 | 596  35.2  49.3  15.4  0.40 | 0.2872  0.1414 | N  GG  GA  AA  MAF | 967  91.5  8.3  0.2  0.04 | 597  91.6  8.2  0.2  0.04 | 0.9841  0.9233 |
| *SCARB1 A350A (rs5888)* | | | | *SCARB1 i9107 (rs4765181)* | | | | *SCARB1 G2S (rs4238001)* | | | |
| N  CC  CT  TT  MAF | 952  45.1  42.3  12.6  0.34 | 596  29.4  51.0  19.6  0.45 | < 0.0001  < 0.0001 | N  GG  GT  TT  MAF | 945  39.9  47.0  13.1  0.37 | 591  31.1  51.3  17.6  0.43 | 0.0009  0.0003 | N  GG  GA  AA  MAF | 959  81.4  17.9  0.6  0.10 | 596  81.2  17.3  1.5  0.10 | 0.2153  0.6112 |
| *SCARB1 i82699 (rs701106)* | | | | *SCARB1 i30026 (rs10846748)* ^1^ | | | | *SCARB1 i19960 (rs3924313)* | | | |
| N  GG  GA  AA  MAF | 960  68.0  28.8  3.2  0.18 | 597  70.2  27.1  2.7  0.16 | 0.6191  0.3282 | N  GG  GA  AA  MAF | 962  26.7  53.0  20.3  0.47 | 597  44.2  43.6  12.2  0.34 | < 0.0001  < 0.0001 | N  GG  GA  AA  MAF | 966  57.5  35.4  7.1  0.25 | 597  49.1  40.5  10.4  0.31 | 0.0025  0.0004 |
| *SCARB1 i51973 (rs61932577)* | | | | *WDTC1 i22835 (rs4460661)* | | | | *WDTC1 i61970 (rs3813790)* | | | |
| N  GG  GA  AA  MAF | 956  85.1  14.6  0.2  0.08 | 596  77.0  21.6  1.3  0.12 | < 0.0001  < 0.0001 | N  TT  TC  CC  MAF | 946  61.5  34.2  4.2  0.21 | 597  77.1  20.6  2.3  0.13 | < 0.0001  < 0.0001 | N  AA  AG  GG  MAF | 946  49.6  41.6  8.8  0.30 | 597  68.7  27.5  3.9  0.18 | < 0.0001  < 0.0001 |
| *ZNF568 i23579 (rs544543)* | | | | *ZNF568 i23072 (rs505717)* | | | |  | | | |
| N AA  AG  GG  MAF | 964  36.8  46.5  16.7  0.40 | 590  38.5  50.0  11.5  0.37 | 0.0195  0.0580 | N  AA  AG  GG  MAF | 962  37.0  46.4  16.6  0.40 | 592  38.7  49.8  11.5  0.36 | 0.0201  0.0577 |  |  |  |  |

*p-values were calculated using Pearson’s chi-square for the genotype and the allele distribution in Puerto Ricans versus non-Hispanic whites. All frequencies are presented as percents from total within the population for each gene.

N=Sample Size, MAF=Minor Allele Frequency

^1^ Genotype distribution is not in Hardy-Weinberg Equilibrium for Puerto Ricans

^2^ Genotype distribution is not in Hardy-Weinberg Equilibrium for non-Hispanic whites
