# Supplementary material for: Disparities in allele frequencies and population differentiation for 101 disease-associated single nucleotide polymorphisms between Puerto Ricans and non-Hispanic whites
Source: BMC Genet. 2009 Aug 14;10:45. doi: 10.1186/1471-2156-10-45 (PMC2734553; doi:10.1186/1471-2156-10-45)
Supplement: Additional file 3 — Predicted minor allele frequencies, and measures of population differentiation for Puerto Ricans and non-Hispanic whites. Predicted minor allele frequencies under an admixture model, and measures of population-specific and between-population differentiation for Puerto Ricans and non-Hispanic whites. [file 1471-2156-10-45-S3.doc]

**Additional file 3: Predicted minor allele frequencies, and measures of population differentiation for Puerto Ricans and non-Hispanic whites.**

Description: Predicted minor allele frequencies under an admixture model for Puerto Ricans, and measures of population-specific and between-population differentiation for Puerto Ricans and non-Hispanic whites.

| ***Gene and SNP*** | | ***rs number*** | ***Predicted MAF*** | ***P-value**** | ***F_ST_ Puerto Rican*** | ***F_ST_ NHW*** | ***Between F_ST_*** |
| --- | --- | --- | --- | --- | --- | --- | --- |
| *ABCA1* | K219R | rs2230806 | 0.32 | <0.0001 | -0.006 | 0.238 | 0.088 |
|  | i125970 | rs2297404 | 0.05 | 0.8220 | 0.054 | -0.151 | -0.025 |
|  | i48168 | rs4149272 | 0.41 | 0.0108 | 0.002 | 0.116 | 0.046 |
|  | i27943 | rs2575875 | 0.36 | 0.2770 | 0.002 | 0.102 | 0.040 |
|  | 3U8995 | rs363717 | 0.14 | <0.0001 | -0.085 | 0.346 | 0.083 |
| *ABCG5* | i18429 | rs4148189 | 0.23 | 0.0100 | -0.070 | 0.476 | 0.140 |
|  | m216 | rs3806471 |  |  | 0.013 | -0.032 | -0.004 |
|  | Q604E | rs6720173 | 0.21 | 0.0013 | -0.042 | 0.175 | 0.041 |
|  | i7892 | rs4131229 | 0.25 | 0.0001 | 0.064 | -0.069 | 0.013 |
| *ABCG8* | C54Y | rs4148211 | 0.25 | 0.0126 | 0.065 | -0.089 | 0.006 |
|  | T400K | rs4148217 | 0.23 | 0.5104 | -0.029 | 0.096 | 0.019 |
|  | D19H | rs11887534 | 0.06 | 0.4012 | -0.061 | 0.186 | 0.034 |
|  | i14222 | rs6709904 | 0.16 | 0.0690 | -0.091 | 0.354 | 0.081 |
| *APOA1* | m3012 | rs11216158 |  |  | -0.043 | 0.140 | 0.027 |
|  | m75 | rs670 |  |  | 0.001 | -0.005 | -0.001 |
|  | m2803 | rs2727784 |  |  | 0.030 | 0.095 | 0.055 |
|  | m2630 | rs613808 |  |  | 0.034 | 0.180 | 0.090 |
| *APOA2* | m265 | rs5082 | 0.30 | 0.2794 | 0.036 | -0.059 | 0.000 |
| *APOA4* | A4-A5 Intergenic | rs1263177 |  |  | 0.002 | -0.008 | -0.002 |
|  | N147S | rs5104 |  |  | -0.040 | 0.134 | 0.027 |
|  | S367T | rs675 | 0.14 | 0.3097 | 0.056 | -0.138 | -0.019 |
|  | T29T | rs5092 |  |  | 0.000 | -0.002 | -0.001 |
|  | Q380H | rs5110 | 0.04 | 0.0391 | 0.152 | -0.400 | -0.060 |
|  | m35 | rs5090 |  |  | 0.290 | -0.779 | -0.120 |
| *APOA5* | m1123 | rs662799 | 0.06 | <0.0001 | -0.117 | 0.443 | 0.096 |
|  | S16W | rs3135506 | 0.06 | <0.0001 | -0.108 | 0.358 | 0.072 |
| *APOB* | A618V | rs679899 | 0.30 | 0.2012 | 0.127 | -0.071 | 0.051 |
|  | m516 | rs934197 |  |  | -0.006 | 0.019 | 0.004 |
|  | E4181K | rs1042031 | 0.16 | 0.0283 | 0.049 | -0.132 | -0.020 |
|  | P2739L | rs676210 | 0.16 | 0.0172 | 0.032 | -0.083 | -0.012 |
|  | T2515T | rs693 | 0.34 | 0.0001 | 0.037 | -0.007 | 0.020 |
| *APOC3* | G34G | rs4520 |  |  | -0.015 | 0.051 | 0.010 |
|  | 3U386 | rs5128 |  |  | -0.063 | 0.532 | 0.168 |
|  | m2886 | rs2542051 |  |  | 0.001 | 0.056 | 0.022 |
|  | m640 | rs2542052 |  |  | 0.006 | 0.057 | 0.026 |
|  | m455 | rs2854116 |  |  | 0.007 | 0.061 | 0.028 |
|  | m482 | rs2854117 |  |  | 0.007 | 0.214 | 0.086 |
| *APOE* | m226 | rs405509 | 0.36 | <0.0001 | 0.012 | -0.009 | 0.004 |
|  | R176C | rs7412 |  |  | 0.113 | -0.300 | -0.047 |
|  | C130R | rs429358 |  |  | 0.050 | -0.131 | -0.020 |
| *ATF6* | i190554 | rs2499856 | 0.07 | 0.0385 | 0.081 | -0.226 | -0.036 |
| *CRP* | 3U2131 | rs1205 | 0.25 | <0.0001 | -0.003 | 0.007 | 0.001 |
|  | i178 | rs1417938 | 0.21 | 0.0002 | 0.041 | -0.088 | -0.009 |
| *CYP7A1* | i6782 | rs11786580 | 0.19 | 0.1901 | 0.035 | -0.092 | -0.014 |
|  | Intergenic 3U12536 | rs10957056 |  |  | 0.023 | -0.065 | -0.011 |
| *FABP1* | T94A | rs2241883 | 0.22 | <0.0001 | 0.030 | -0.067 | -0.007 |
|  | m2353 | rs3891700 |  |  | -0.028 | 0.139 | 0.033 |
| *FABP2* | A55S | rs1799883 | 0.26 | 0.0795 | -0.008 | 0.020 | 0.003 |
|  | m193 | rs6857641 |  |  | 0.003 | -0.010 | -0.002 |
|  | m767 | rs10034661 | 0.29 | 0.0301 | -0.019 | 0.061 | 0.012 |
| *GCKR* | i21532 | rs780094 | 0.26 | 0.0257 | 0.057 | -0.079 | 0.003 |
| *LIPC* | i33753 | rs7169744 | 0.11 | 0.2232 | 0.039 | -0.112 | -0.018 |
|  | V95M | rs6078 | 0.01 | <0.0001 | -0.153 | 0.476 | 0.089 |
|  | T224T | rs6084 | 0.36 | <0.0001 | 0.018 | 0.024 | 0.020 |
|  | i618 | rs8034802 | 0.27 | <0.0001 | -0.022 | 0.104 | 0.027 |
|  | i67180 | rs1973028 | 0.38 | 0.0001 | -0.005 | 0.052 | 0.017 |
| *LIPG* | i13576 | rs2276269 | 0.29 | <0.0001 | 0.009 | -0.010 | 0.001 |
|  | i24582 | rs6507931 | 0.48 | 0.1371 | 0.000 | 0.002 | 0.001 |
|  | T111I | rs2000813 | 0.19 | 0.0005 | 0.010 | -0.030 | -0.005 |
| *LPL* | D9N (D36N) | rs1801177 | 0.02 | 0.0291 | -0.162 | 0.505 | 0.093 |
|  | N291S (N318S) | rs268 | 0.01 | 0.0010 | -0.146 | 0.449 | 0.081 |
|  | S447X (S474X) | rs328 | 0.09 | 0.1182 | -0.009 | 0.023 | 0.003 |
|  | m107 (m93) | rs1800590 | 0.15 | 0.0695 | -0.192 | 0.826 | 0.198 |
| *LRP1* | i10701 | rs715948 | 0.24 | 0.0983 | 0.028 | -0.066 | -0.008 |
|  | C766T | rs1799986 | 0.11 | 0.0814 | 0.089 | -0.225 | -0.032 |
|  | i68477 | rs1800191 |  |  | -0.012 | 0.072 | 0.020 |
| *MTTP* | C174C | rs982424 | 0.10 | 0.6301 | -0.083 | 0.266 | 0.051 |
|  | i10249 | rs1800591 |  |  | -0.030 | 0.114 | 0.025 |
|  | i9314 | rs3811800 |  |  | -0.011 | 0.121 | 0.040 |
| *NOS3* | m459 | rs11771443 |  |  | -0.051 | 0.193 | 0.043 |
|  | i19342 | rs743507 | 0.18 | 0.0025 | 0.024 | -0.064 | -0.010 |
|  | i1103 | rs1800783 | 0.35 | 0.0038 | -0.003 | 0.008 | 0.001 |
|  | E298D | rs1799983 | 0.22 | 0.1061 | 0.053 | -0.108 | -0.009 |
| *PDZK1* | i19738 | rs1284300 | 0.06 | 0.0072 | 0.019 | -0.056 | -0.010 |
| *PLIN* | 3U2197 (PLIN6) | rs1052700 | 0.20 | 0.1422 | 0.112 | -0.191 | 0.001 |
|  | i10769 (PLIN4) | rs894160 | 0.30 | 0.0288 | -0.003 | 0.006 | 0.001 |
|  | i5496 (PLIN1) | rs2289487 | 0.40 | <0.0001 | 0.024 | 0.138 | 0.068 |
|  | P371P (PLIN5) | rs2304795 |  |  | -0.004 | 0.010 | 0.002 |
| *PPARA* | L162V | rs1800206 | 0.03 | <0.0001 | 0.009 | -0.027 | -0.005 |
|  | i5522 | rs135549 | 0.47 | 0.0729 | 0.001 | 0.008 | 0.004 |
| *PPARG* | H477H | rs3856806 | 0.07 | 0.6535 | 0.177 | -0.428 | -0.055 |
|  | m39803 | rs10865710 | 0.18 | 0.0001 | 0.028 | -0.071 | -0.010 |
|  | m2866 | rs12497191 | 0.09 | 0.0015 | 0.008 | -0.026 | -0.005 |
|  | P12A | rs1801282 | 0.05 | 0.0986 | 0.255 | -0.567 | -0.061 |
| *PPARGC1A* | i27289 | rs4235308 | 0.36 | 0.0302 | -0.003 | 0.006 | 0.001 |
|  | i5378 | rs2946385 | 0.42 | 0..4923 | -0.004 | 0.017 | 0.004 |
|  | m1668 | rs2970869 | 0.16 | <0.0001 | -0.022 | 0.076 | 0.016 |
|  | T612M | rs3736265 | 0.07 | 0.2635 | -0.043 | 0.130 | 0.023 |
|  | i55301 | rs4697046 | 0.36 | 0.7374 | 0.007 | -0.018 | -0.002 |
|  | 3U4898 | rs3774923 | 0.06 | 0.0074 | -0.004 | 0.011 | 0.001 |
| *SCARB1* | A350A | rs5888 | 0.34 | 0.8372 | 0.053 | -0.049 | 0.014 |
|  | i9107 | rs4765181 | 0.28 | <0.0001 | 0.023 | -0.033 | 0.001 |
|  | G2S | rs4238001 |  |  | 0.013 | -0.039 | -0.007 |
|  | i82699 | rs701106 | 0.16 | 0.1578 | -0.016 | 0.047 | 0.008 |
|  | i30026 | rs10846748 | 0.36 | <0.0001 | 0.007 | 0.105 | 0.045 |
|  | i19960 | rs3924313 | 0.28 | 0.0677 | 0.041 | -0.092 | -0.010 |
|  | i51973 | rs61932577 |  |  | 0.131 | -0.333 | -0.047 |
| *WDTC1* | i22835 | rs4460661 | 0.20 | 0.5465 | -0.072 | 0.294 | 0.070 |
|  | i61970 | rs3813790 | 0.26 | 0.0066 | -0.048 | 0.271 | 0.076 |
| *ZNF568* | i23579 | rs544543 | 0.32 | <0.0001 | -0.006 | 0.027 | 0.006 |
|  | i23072 | rs505717 | 0.32 | <0.0001 | -0.007 | 0.027 | 0.006 |
| Overall |  |  |  |  | 0.008 | 0.039 | 0.020 |

*P-value shown for chi-square test of the differences between the predicted MAF under an admixture model and the observed MAF in the Puerto Rican sample. Empty cells indicate SNPs without available HapMap information for calculations of predicted MAF.
